# Governing health through security in the Philippines: a realist analysis

**DOI:** 10.1093/heapol/czaf110

**Published:** 2025-12-12

**Authors:** Delaram Akhavein, Lea Elora A Conda, Sary Valenzuela, Percival Ethan Lao, Meru Sheel, Seye Abimbola, Geminn Louis C Apostol

**Affiliations:** School of Public Health, Faculty of Medicine and Health, The University of Sydney, Edward Ford Building, Sydney, NSW 2006, Australia; School of Medicine and Public Health, Ateneo de Manila University, Don Eugenio Lopez Sr. Medical Complex, Ortigas Ave, Pasig 1604, Philippines; School of Medicine and Public Health, Ateneo de Manila University, Don Eugenio Lopez Sr. Medical Complex, Ortigas Ave, Pasig 1604, Philippines; Department of Global Human Development, Georgetown University, 3700 O St NW, Washington, DC 20057, United States; School of Medicine and Public Health, Ateneo de Manila University, Don Eugenio Lopez Sr. Medical Complex, Ortigas Ave, Pasig 1604, Philippines; School of Public Health, Faculty of Medicine and Health, The University of Sydney, Edward Ford Building, Sydney, NSW 2006, Australia; School of Public Health, Faculty of Medicine and Health, The University of Sydney, Edward Ford Building, Sydney, NSW 2006, Australia; School of Medicine and Public Health, Ateneo de Manila University, Don Eugenio Lopez Sr. Medical Complex, Ortigas Ave, Pasig 1604, Philippines; College of Healthcare Sciences, James Cook University, Townsville, Queensland 4811, Australia

**Keywords:** health securitization, health security, global health, Philippines, decentralization, devolution, governance, health systems

## Abstract

As global framings of health continue to expand their reach, the Philippines, like many other countries, must navigate the overlapping pressures of donors, international institutions, and domestic political agendas in setting priorities. One such framing is the framing of health as a security issue. This study examines how health security framing—how it is interpreted and operationalized—influences priority-setting in the Philippines. Drawing on 25 interviews with government (at national and sub-national levels) and non-government actors and using a realist approach, this study sought to identify the outcomes of health security framing (as triggered or reinforced by mechanisms such as uncertainty, self-protection, self-preservation, self-reliance, and norm-setting) and the context in which the outcomes manifest. Findings show that health security framing reshapes priorities by reinforcing centralized, top-down approaches at both international and national levels. These framings influence not only what is prioritized but also which actors make decisions and how those decisions are justified. At the implementation level, it manifests in health workers facing misaligned operational frameworks, vertical programming, and burdensome reporting requirements tied to donor funding. Security norms become institutionalized with the involvement of military and security actors in health. The study demonstrates that health security is not a static concept, but a dynamic phenomenon co-constructed through global discourses, donor agendas, and domestic governance practices, all of which are shaped by power relations and history. While health security mobilizes resources and political attention, it also introduces trade-offs that risk exacerbating inequities and diverting attention from the structural determinants of health.

Key Messages:

- While health security is often framed as a technical or humanitarian goal, it is shaped (intentionally or unintentionally) by geopolitical interests and donor priorities. In the Philippines, it can become a tool for portraying national competence and attracting global investment.
- Framing health as a security issue drives centralization of power both within countries and internationally. Securitized health policies favour surveillance and vertical programs, which struggle to integrate into the health system.
- Surveillance and reporting systems are shaped by the need to show competence and meet global benchmarks, while acknowledging that there are political dimensions to sharing data.
- The framing of health as security legitimizes the role of the security sector actors in public health governance.

## INTRODUCTION

In recent years, the Philippines has increasingly engaged with health security frameworks, navigating a complex landscape shaped by international norms, donor priorities, and domestic health system challenges (World Health Organization 2025a, 2025b). However, what exactly is Health Security? Who is it for, and what does it mean for the Philippines? The growing literature on the meaning of this concept and its applications remains ambiguous, revealing not only a multitude of perspectives but also various ways it can be operationalized within health systems (Aldis 2008, Rushton 2011, Malik et al. 2021). Predominantly used in relation to the control of, and response to infectious diseases such as Ebola, Zika and most recently, and perhaps the most relevant to the Philippine context, COVID-19 (Heymann et al. 2015, Gostin and Hodge 2016, Tsukayama et al. 2020, Pannu and Barry 2021), its use continues to grow across the broader field of ‘Global Health’ (Ollila 2005, Holst and Razum 2022).

The World Health Organization (WHO) defines health security—primarily used and understood by those responsible for its downstream implementation—as the ‘activities required, both proactive and reactive, to minimize the danger and impact of acute public health events that endanger people’s health across geographical regions and international boundaries’ (World Health Organization 2025a, 2025b). However, this definition (whether intentionally or otherwise) underplays several ambiguities (Voss et al. 2022). On one hand, health security is used and understood alongside or as part of the field of public health, to bring into focus aspects that help control and minimize the impacts of public health events, as the definition states. On the other hand, the intersection of health with security introduces implications associated with ‘securitization’ (Buzan et al. 1998) and notions of ‘state-centric maintenance of international peace and security’ (Voss et al. 2022). Here, we take health securitization as the broader political and discursive process that produces health security as a policy and framework. Within this framing, certain health events are perceived as existential threats to states requiring exceptional measures beyond normal political processes (Buzan et al. 1998, Ingram 2005), which may include surveillance, border control, emergency powers, or military intervention. Such framing is done to mobilize resources, institutions, and public support to address the perceived threat and protect the security and survival of a society or state (Ingram 2005, Fidler 2007). On closer examination, it may be noted that the WHO’s formal definition of health security, in fact, describes such a desired outcome—one that is achieved through the *process* of securitization (Voss et al. 2022). While securitization facilitates resource mobilization and draws political attention, (Gerami et al. 2021, Mohd Azmi 2020), it is worth exploring whether it does so with longer-term trade-offs, particularly in its potential to reshape health system priorities and governance structures.

As the health security agenda becomes increasingly embedded in the ways international organizations like the WHO and the United States’ Centre for Disease Control and Prevention (CDC)—particularly through the promotion and use of International Health Regulations (IHR)-related tools. One example is the Joint External Evaluation (JEE), which was developed to assess countries’ ability to respond to ‘health security concerns’ and to inform the development of country National Action Plans for Health Security (NAPHS). Against this backdrop, there is a growing need to understand how this framing influences health system governance and practices, as well as the ways in which priorities are set (Kentikelenis and Seabrooke 2022, Hayman et al. 2023, Centers for Disease Control and Prevention 2024). It involves understanding the political and institutional dimensions, including issues of ownership, local relevance, and the specific contexts in which such a framework operates.

In the Philippines, the health system is composed of both the public and private sectors, with most health services delivered by private providers (Grundy et al. 2003, Cuenca 2018), while governance, financing, and regulatory oversight lie primarily with the public sector. However, the responsibility for financing, management and service delivery is devolved to Local Government Units (LGUs), while the Department of Health (DOH) retains primary responsibility for technical guidance and regulatory oversight functions. This devolution arrangement was designed to enhance efficiency and foster participatory local governance. Tension between national oversight and local autonomy remains a core feature of the Philippine health system, which was most recently visible during the COVID-19 pandemic (Griffith Asia Institute 2020, Gomez-Chua et al. 2023) when the national-level responses framed the pandemic as a matter of health security, invoking language and strategies consistent with global frameworks, leaving implementation with limited resources and competing mandates at the level of the LGUs (Griffith Asia Institute 2020, Hapal 2021, Gomez-Chua et al. 2023).

**Figure 1:**
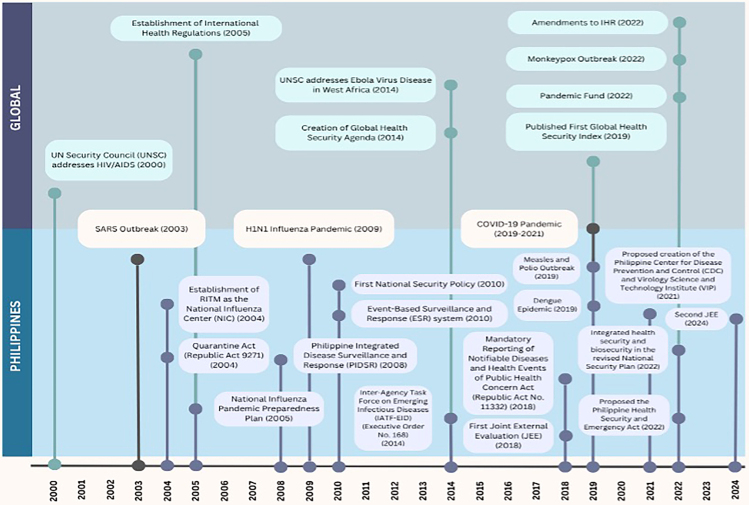
The evolution of ‘health security’ in the Philippines (adapted from Rethinking Health Security After COVID-19) (Gerami et al. 2021).

The Philippines frequently faces health emergencies ranging from typhoons to disease outbreaks (United Nations in the Philippines 2024), and the evolution and adoption of health security frameworks can be seen as both a strategic necessity and a reflection of international influence (Fig. 1; Gerami et al. 2021). While the language of health security has helped mobilize external funding and technical support, it has also introduced new pressures: aligning national health planning with external evaluations, meeting donor-defined benchmarks, and projecting an image of readiness and competence. This dynamic is not new; security-based framings often serve to legitimize state action and international engagement while depoliticizing the underlying drivers of vulnerability or exclusion (Reid 2011, 2016). In this sense, securitization may not only obscure the complexity of health systems and the rationale for health system priority-setting but also embed hierarchies of power between donors and recipients, national and local governments, the security sector and the health sector, technical experts and implementers (Holst 2020).

These dynamics raise questions about how the concept of health security is interpreted and operationalized by country actors, and how it interacts with existing health system priorities and capacities in the Philippines. In this study, we explore the consequences of how health security is understood, interpreted, and operationalized in the Philippines. We aimed to better understand the dynamics that influence how health security framing is translated into practice, and how these processes affect health system priorities (including governance and service delivery). To achieve this, we adopted a realist approach, which is useful for examining complex social phenomena such as the securitization of health. We sought to uncover how and under what conditions the causal mechanisms at play (in this case, the mechanisms that trigger or reinforce the securitization of health) generate outcomes, asking not only what happens but also for whom, and under what circumstances(Pawson and Tilley 1997, Pawson et al. 2004).

## METHODS

### Theoretical framing

**Figure 2:**
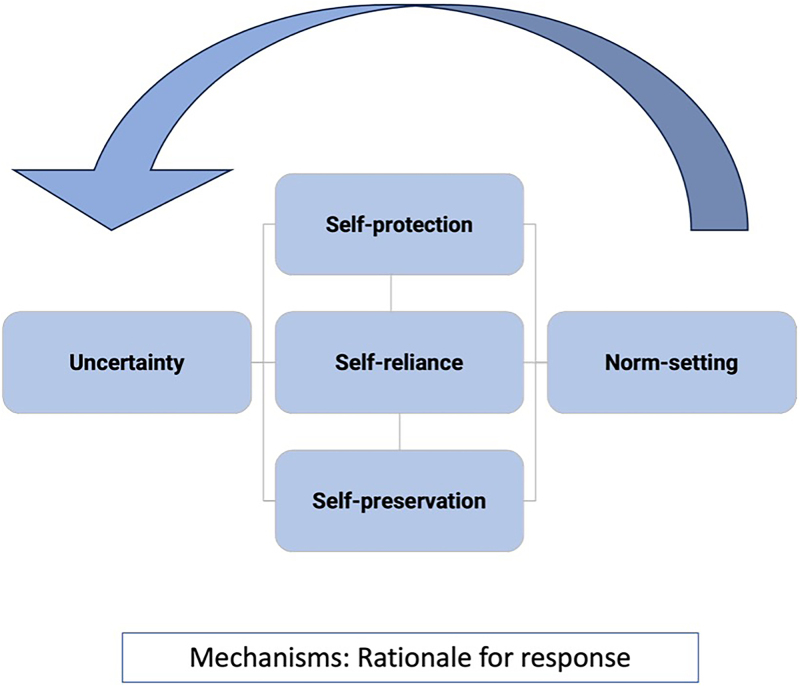
An illustration of the five conceptual mechanisms through which health security framing is enacted. Adapted from Akhavein et al. 2025a.

With the goal of refining theory, we applied an analytical framework from a previously published realist synthesis (Akhavein et al. 2025a). The framework conceptualizes five mechanisms (Fig. 2) through which the process of (health) securitization or the application and use of health security is activated, reinforced, or maintained as rationale for action—‘uncertainty’ (the ambiguity of risks), ‘self-protection’ (safeguarding own group/national interests), ‘self-reliance’ (ability to be autonomous), ‘self-preservation’ (maintaining own institutional credibility), and ‘norm-setting’ (establishing long-term policy shifts) (Fig. 2 and Table 1). We used this framework to analyse how aspects of context activate or deactivate the generative powers of these mechanisms in the Philippines, resulting in specific outcomes. This means that a given mechanism may not produce the same outcome in different contextual circumstances nor that an outcome is only generated by or observable through that mechanism. Additionally, a mechanism active at one level of the system can create conditions for the same or another mechanism to unfold at another level. As contexts shift and actors move between different governance levels, various mechanisms may be triggered. In other words, the same actor may be driven by different mechanisms depending on the context in which the actor is operating.

**Table 1:** Mechanisms through which health security is triggered or reinforced.

Mechanism	Conceptual definitions
Uncertainty	Ambiguity about risks create justification for external guidance and donor control
Self-protection	Actors seek to safeguard state or political interests from perceived threats
Self-preservation	Maintaining institutional or professional credibility
Self-reliance	Projecting autonomy
Norm-setting	Institutionalizing frameworks into routines and standards

**Figure 3:**
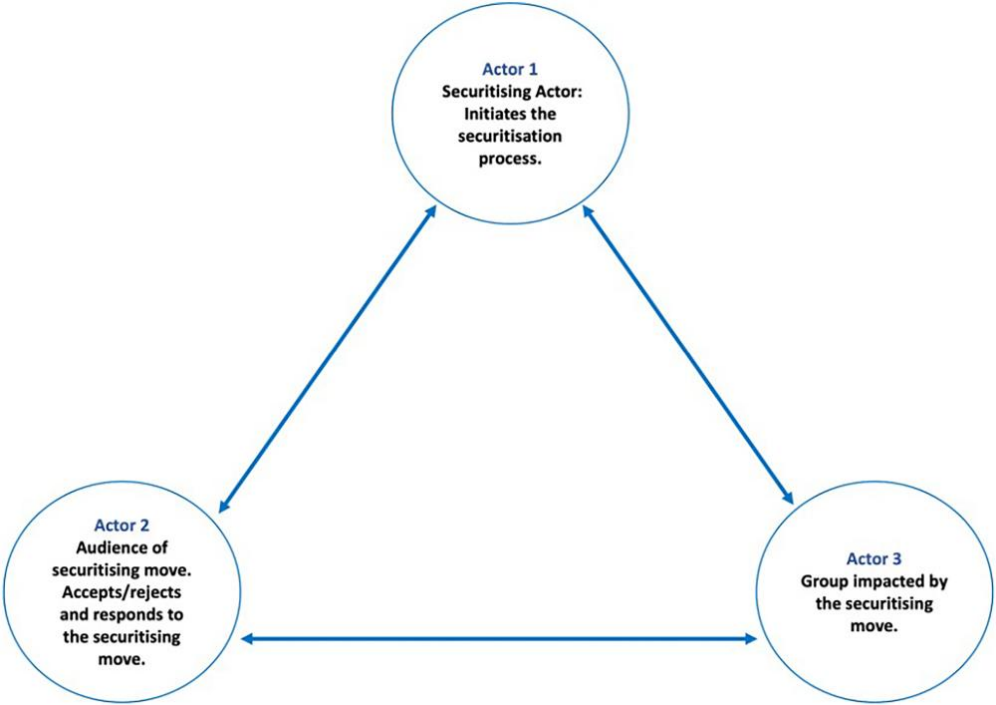
An illustration of the three-actor interactions. Adapted from Akhavein et al. 2025a, 2025b.

The framework of five mechanisms (Akhavein et al. 2025a) was developed alongside, the Securitization Theory (Buzan et al. 1998), which we used to examine how health security was mobilized as a discourse and operationalized in practice. In this framework, we extended the original two-actor model presented by Buzan *et al.* to include a third group of actors, thereby creating a three-actor analytical framework (Fig. 3). Actor 1 represents the securitizing actor, initiating the securitization process (e.g. government agencies, security institutions, or international funders). Actor 2 represents the audience, typically those in a position to legitimise or accept the securitizing move (e.g. sub-national implementers, healthcare managers). However, finally, Actor 3 denotes those most affected by the consequences of securitization (e.g. communities, frontline healthcare workers). This actor framework helped us map how securitizing practices were initiated, received, and experienced at different levels of the Philippine health system (Akhavein et al. 2025b).

### Participants and sampling

This qualitative study is reported following the COREQ (Consolidated Criteria for Reporting Qualitative Research) 32-item checklist (Tong et al. 2007). The completed checklist is provided in Supplementary File S1, where additional information can also be found. Participants were recruited through purposive and snowball sampling from a broad range of sectors engaged with health security frameworks and policies in the Philippines (Table 2). These included officials from Departments of Health at both national and sub-national levels, the National Security Council and the Department of National Defence, healthcare workers, and stakeholders from international organizations and funding agencies.

**Table 2:** Summary of the characteristics of interviewees for this study.

No. of interviewees	25
Male	15
Female	10
Category of current roles	
DOH Division Chiefs/Department Heads	7
DOH Regional Director	1
DOH Regional Office Program Managers/Heads	5
Provincial Officers	5
Head of Military Health Service/National Security	2
Head of an NGO	1
Employee of an NGO	2
Independent Consultant	2
Organizational affiliation at the time of the interview	
National Agencies (e.g. DOH, BOQ, DA)	7
Regional Offices	6
Provincial Offices	5
Military-related Facilities/Councils/Agencies	2
NGOs	5
Level of decision-making at the time of the interview	
National Level	9
Regional Level	6
Provincial Level	5
Not applicable	5
Geographic focus of role at the time of the interview	
Nationwide	14
Luzon	2
Visayas	7
Mindanao	2

The Philippines is an archipelago divided into Luzon, Visayas, and Mindanao, which is made up of 18 administrative regions. In each region, a city is designated as the centre where a regional office of the national government is housed. The island topography means that there are regional disparities, specifically in municipalities and cities in Visayas and Mindanao, as a result of physical isolation and political barriers to resources and governance capacity (Leyso and Umezaki 2024). While we sought to include regionally diverse insights, the primary objective was to ensure representation across levels of governance and institutional roles and to include actors from agencies with a clear connection to the planning and implementation of initiatives related to ‘health security’ (Table 2). In total, seven regions were represented. Particular attention was given to ensuring the inclusion of sub-national actors, as the research sought to test the proposition that many of the observable ‘outcomes’ of health security practices are also experienced at the local level. Although the general population or health service users may also be conceptualized as Actor 3, the impact on this particular group was inferred in this study.

### Data collection

Qualitative data were collected using realist interviews. Realist interviewing is a theory-driven approach to interviewing that enables the uncovering of causal pathways and the explanation of how outcomes arise in specific contexts. A key distinction between realist interviews and other interview techniques is the presentation of theory to respondents (Westhorp and Manzano 2017, Mukumbang et al. 2020). This approach is particularly well-suited for policy-relevant research that aims to uncover complex phenomena. In this study, realist interviews offered a way to explore not only what was happening in relation to the planning and implementation of initiatives related to ‘health security’ in the Philippines but also the propositions of why, how, and for whom particular dynamics unfolded in those initiatives. The theory-informed nature of realist interviews made it possible to introduce and probe abstract concepts, such as health security, global frameworks, governance tensions, and candidate causal explanations (using the five mechanisms), throughout the interview process (Mingers and Standing 2017, Fryer 2020). Interviews were guided by the preliminary context-mechanism-outcome (CMO) configurations that were developed from an earlier realist synthesis (Akhavein et al. 2025a), which allowed respondents to reflect on their experiences while helping to refine and contextualize the initial theories and explore their relevance empirically. The ‘outcome’ was framed in terms of how the implementation or manifestation of health security policies and practices may affect prioritization in the health system, and by extension, health service delivery. The interviews focused on participants’ experiences and interpretations in relation to specific events, decisions, or strategies. All interviews were pseudonymized.

### Data analysis and synthesis

We adopted the realist CMO heuristic to examine how the underlying mechanisms that trigger or reinforce health security discourses and practices influence health system priorities (outcomes) in the Philippines (Pawson and Tilley 1997). In this study, we employ realism as an epistemological concept, rather than realism as applied in international relations scholarship, which focuses on state behaviour and material self-interest (Wohlforth 2008). While our analysis acknowledges that material and strategic interests can themselves serve as mechanisms that trigger or reinforce health security practices (e.g. the mechanism of self-protection), these are analytically distinct from the realist methodological framework we employ in our study (Pawson and Tilley 1997).

Data analysis began with immersive readings of all transcripts to gain familiarity with the language, tone, and ways in which participants articulated and interpreted their experiences. This allowed for a better understanding of how health security and related concepts were discussed before applying the CMO analytical framework (Jagosh 2019). Subsequently, the entire research team contributed to the conceptualization of the analytical framework, after which the transcripts were manually coded using a customized Excel spreadsheet against five pre-identified mechanisms—drawn from an earlier realist synthesis (Akhavein et al. 2025a). Rows represented individual respondents, with five columns corresponding to the five mechanisms. Additional columns were created for ‘context’ and ‘outcome’, populated whenever participants explicitly or implicitly referred to these dimensions. Verbatim excerpts were entered into the cells where participants’ narratives reflected, implied, or illustrated a particular mechanism. Given the complexity of the narratives, a single transcript often demonstrated more than one mechanism simultaneously. Contexts and outcomes were refined through repeated readings, comparisons across transcripts, and iterative categorization until a coherent and representative set of outcomes was developed. Throughout the data analysis and categorization process, multiple online discussions were held with the team to validate and verify categories and themes. The sets of outcomes informed the structure of the findings section (Table 3). CMO linkages were not derived from single interviews alone; instead, they were built by synthesizing insights from data across multiple transcripts, recognizing that participants often described parts of a causal chain that needed to be analytically assembled.

**Table 3:** Conceptual definitions of health security outcomes and the role of actors.

Outcome	Actor 1	Actor 2	Actor 3
	Securitizing actor	Audience	Group most affected
Governance centralization: refers to the concentration of authority and decision-making power to higher, external structures. External does not necessarily refer to ‘foreign’ but rather to external bodies to that of the governance level in question—e.g. international to national to subnational.	WHO, donor states, funding agencies	National government	Sub-national governments, healthcare workers, communities
	National government	Sub-national government, military/security	Local implementers, communities
Shaping resource allocation: refers to the actions taken at the policy and decision-making level. This is seen as the next step to centralization—the type of policies that are set and where prioritisation happens. It does not specifically refer to monetary allocation of resources but also of technical expertise and health service delivery workforce	WHO, Philanthropic agencies, bilateral donors	National/sub-national, donor-funded program staff	healthcare workers, facility managers, patients
	National government	Sub-national government, technical staff	Local health workers, communities
Reconfiguring health services: refers to the translation from policy to practice with varying effects on health service delivery at the implementation level. These impacts, which are downstream effects of shaping allocation of resources, influence both the delivery of care and the conditions under which health workers operate.	Donors, technical experts, DOH	Sub-national government, INGOs	HCWs, local implementers
	INGOs (as programme designers)	Healthcare workers (programme implementers)	Service users/patients
Institutionalizing security norms: refers to how the use of security language, and the conflation of health with security influenced by political ideologies, shape ‘threat’ identification, and the involvement of security actors in health.	WHO, donor states. National Defence Agencies	Military, national government, elite groups	Civil societies, communities
	Academics, National government	Academic institutions, military units	Healthcare workers, communities

### Ethics

The Research Ethics Office of the authors’ institute has provided ethics approval for this study. Participation in the study was entirely voluntary and based upon the participant signing a written informed consent form after receiving an information sheet with details about the study and their involvement. In line with the terms of consent to which participants agreed, the data for this study are not publicly available, and all participants have been de-identified by removing potentially identifying information.

## FINDINGS

Table 2 presents a summary of the characteristics of the (*n* = 25) interviewees; a group of stakeholders engaged in health governance and programming in the Philippines. The participant roles spanned decision-making at the national, regional, and provincial levels, as well as program implementation and technical functions, with geographic responsibilities ranging from nationwide to Luzon, Visayas, and Mindanao.

The findings are presented in four sections that reflect how health security framing influences governance and practice in the Philippines: governance centralization, shaping of resource allocation, reconfiguring health services, and institutionalization of security norms (Table 3). Governance centralization reflects how authority and decision-making are pulled upward, shaping resource allocation, resource allocation refers to how policies and priorities are set, reconfiguring health services captures how these policy choices are translated into practice at the level of service delivery, and finally, institutionalizing security norms illustrates how health becomes entangled with security discourses and institutions. Across all four outcomes, different actors interact in shifting roles—sometimes as securitizing actors (Actor 1), sometimes as audiences (Actor 2), and sometimes as those most affected (Actor 3). For example, national governments (Actor 2) may adopt donor (Actor 1) framings but also reinterpret and enforce them, shifting between Actor 2 and Actor 1, while frontline workers (Actor 3) may experience this through administrative burdens and conditionalities. There is a fluidity and a non-linearity in how health security is imposed and adopted (Fig. 2).

### Governance centralization

At the international level, centralization of governance may result from geopolitical **self-protection** and consequent **norm-setting** when global health institutions and donor states centralize governance to exert influence, shape policy frameworks, set the terms of debate, and steer decision-making processes that cascade down to national and subnational levels. Such international centralization may result in misalignment between international agendas and local priorities, when, although ‘The local health context in our country [Philippines] can be totally different from that of our neighbouring Asian countries’ (GA-4), such that countries are treated as though they are the same. Centralization at the international level also reaches through the national level to the sub-national level in how global institutions and donor states’ funding is allocated—‘Somebody from the central [national] office came to my[sub-national] office because they wanted to create a One Health Laboratory. I asked them, What's the objective? We already have our research institute for tropical medicine …And then they were saying, we need to have this because by 2025, the grant from the government of the United States and other countries are about to end’ (SGA-4). Enabled by technical and financial support, international centralization may manifest as top-down influence on sensemaking, ‘I'm not very familiar with the politics of all of this. But every time we go to Geneva…one strong entity is the US, and we notice that some of our [Philippines] thinking were largely influenced by their kinds of thinking…given that the US involvement in our technical work, may be a way to influence, you know, their full stronghold…’ (SGA-5)—which may disrupt local priorities and domestic planning—‘I think globally, the discussions are being shaped by the nations that have more political clout or countries with influence in terms of funding …these countries can dictate what kind of priorities we should focus on’ (SGA-9). Enabled by such a context of imbalances of power and resources, the priorities of a powerful country and its agents operating in a **self-protection** mode may impact national prioritization at a less powerful or influential country level: ‘Driven by self-interest, wherein you know, we see that a lot of the diseases that are being dictated by the global health security agenda, these are not the most pressing issues in our own country’ (ACGA-1).

Influenced by such international centralization, national governments replicate this centralizing logic within national governance structures; an outcome of **uncertainty** and the national government’s **self-preservation**. Despite formal devolution in the country, participants suggested health emergencies trigger a recentralization of previously decentralized governance structures. This recentralization is enabled by a context in which the national government, through the DOH under the Office of the President, retains significant authority in shaping health priorities and decision-making sub-nationally. According to a sub-national level government actor, ‘The prioritization depends also on who's sitting in, let's say in each central office, because, for example, where I work, we do not work directly with the central office. We are a distinct agency from the Department of Health. So, in our experience, priorities change with the one sitting at the top, I mean, every time we have a new Secretary of Health, he or she would have his or her own priority agenda and therefore our prioritization also changes’ (GA-3). Enabled by LGUs’ dependence on the DOH for strategic direction and funding, centralization manifests in the implementation of nationwide policies that are not well-adapted to sub-national peculiarities. Although LGUs are meant to operate autonomously, during emergencies and crises, the national government exerts centralized control and top-down decision-making on LGUs, an apparent outcome of the desire to display or project **self-reliance**, and of **uncertainty**, where, with limited time and clarity, national governments default to ‘command-and-control’ approaches. This was described as generating confusion and frustration among LGU officials, when national priorities do not reflect local needs, capacities, or epidemiological realities -‘Most of the time, they [national government] will be laying out that these are the programmes that need to be implemented in the entire country, but sometimes it's not a reflection of the priorities of the regional office or of the local government’ (SGA-4).

National centralization may also be motivated by **self-preservation**, in contexts of heightened political scrutiny where consolidating authority allows central actors to maintain control over resources, narrative, and policy direction. Political appointees, whose positions may be contingent on perceived performance or loyalty to the President, are particularly motivated to demonstrate decisiveness. Here, **self-preservation** overlaps with **self-reliance**, that is, the national government’s desire to project an image of control—the centralization is the platform in which communication with international organizations often takes place, as a result, it becomes both a political strategy and a performative act. As one participant said, describing the importance of being able to project national—and even departmental—competence—‘Domestic ideologies and priorities matter, and of course, it depends on the administration; not only in the high-level; departmental ideologies also matter’ (NGA-6).

### Shaping resource allocation

In the Philippines, health security’s translation to policy and practice occurs within the political and historical context of being a long-term recipient of foreign aid, particularly from the United States. Established in 1961, ‘USAID has been in the Philippines for a long, long time, more than 60 years’ (NGA-3). With this influence, disease-specific, vertical programs primarily funded by bilateral donors have been prioritized in ways that reflect donor interests rather than locally determined health needs ‘You know, they have the power to be able to influence, since they decide where the money goes and where it should be spent’ (ACGA-1). The geopolitical entanglements of the Philippines are such that regions such as Mindanao being geographically close to the South China Sea, accentuates the **self-protection**-induced U.S. strategic interests and military presence in Southeast Asia, making the Philippines an ‘intensive support partner’: ‘With the Philippines as an intensive support partner, we’re investing on how the Philippines can be better at prevention, detection, and response in health security’ (NGA-3). This external and externally induced investment in strengthening surveillance and response capacities reflects a donor-driven vision of preparedness, described as not necessarily in alignment with domestic health priorities. Notably, non-US actors also engage in health support tied to and informed by the rationale of **self-protection**: ‘They help us with Dengue so that if Koreans go here, they will be protected from that’ (SGA-10)—deployed not only to strengthen bilateral relations but also to ensure the safety of Korean nationals travelling to the Philippines. In addition, during the COVID-19 vaccine rollout, the Philippines struggled to negotiate timely access to vaccines and was often left to accept near-expired doses from donors, with donors working from a position of **self-protection** and **uncertainty**. In this context, vaccination donations, preceded by the provision of personal protective equipment and testing kits, were seen as functioning as a form of soft power—‘During the pandemic, China’s donations to us was used purely to influence the Philippines’ (GA-8)—rather than primarily to address public health needs in the Philippines. Philippine actors themselves, working from a similar position, also engaged in similar practices, offloading unwanted vaccines to other countries, as one respondent put it, ‘The Philippines is also trying to look for other countries who will accept our excess vaccines’ (GA-1).

While international actors often set the agenda and define normative frameworks for health security, the Philippines responded by adopting these frameworks, sometimes strategically, to maintain legitimacy or attract resources. In this sense, **norm-setting** is not a one-way process; the Philippines may adopt the health security framing to align with donor expectations while simultaneously using it to maintain domestic political standing. A significant portion of health security investments in the Philippines—that is, of resource allocation for health—both in terms of time and financial resources, was thus channelled towards fulfilling the **norm-setting** benchmarks outlined in the JEE, which assesses a country’s compliance with the IHR and aligns with the Global Health Security Agenda (GHSA). These evaluations inform the development of the National Action Plan for Health Security (NAPHS), a multi-year roadmap that demands extensive stakeholder engagement and incurs substantial costs. In turn, these externally defined metrics set standards for where efforts should be directed, which creates a pressure to perform or project **self-reliance**—‘You know it's a very honest evaluation. While at the same time, departments have this somehow pressure to score themselves higher so as to be able to look more competent as a country to respond to these health security threats’ (ACGA-1).

Donor agencies and international partners frequently rely on global trends to justify country interventions, but often use country data to enhance the legitimacy of the interventions and consequent resource allocation, which may lead to a perception of misuse or misrepresentation of national data: ‘the data was requested on the guise of preparing the report of another Unit; unfortunately, it turned out that the data shared by our office was used for another purpose’ (GA-6). The participant described such repurposing without consent in terms of undermining trust and cooperation and contributing to scepticism among national institutions, who often feel their data is valued more for its utility in international publications or metrics than for addressing immediate, local health needs: ‘Our data was published in an international paper without our acknowledgement. The data should be used only for the reporting of a target or accomplishment for that particular period’ (GA-6). Such concern also means that, driven by **self-preservation**, ‘[The] DOH might want to have control over the data and which data to share with the international community. This presents a challenge because it provides a longer turnaround time to data sharing’ (GA-9). As a knock-on effect of the national government wanting to take control of country data, some [technical stakeholders] feared that it was at the cost of transparency—‘My fear is that if we discover something new in the Philippines, we will be forced to keep silent’ (GA-9). Data curation is also an issue of resource allocation. National authorities often require detailed subnational data to maintain oversight and control, but responsibility of providing resources to collect, consolidate, and report this data typically falls on subnational units which are generally much less resourced -‘If the Department of Health wants a programme to be implemented, supported by a policy, the more local government units are inclined to comply… the Provincial Office will consolidate the data and submit the reports’

### Reconfiguring health services

International perceptions of risk and threat influence national priorities, including decisions about what is funded, who provides the funding, how funding is structured, and how programs are managed. Using the example of antimicrobial resistance, a participant described how donor **self-preservation** shapes priorities in health service provision: ‘We can see that they are funnelling funds to countries which they think are prone to have the emergence of resistance… such as Thailand and the Philippines… eventually, I think they feel that… this resistant bacteria will reach their shores… So in order to protect their interest, they need to ensure that this health concern is recognized… and manage any resistant pathogens that are detected’ (GA-3). First triggered by **self-preservation**, **norm-setting** reinforces patterns of dependency on externally driven funding agendas. Conditionalities, a feature of foreign assistance, was described by one respondent as: ‘There are non-negotiables, they would say they would do all this, but they sometimes have those negative lists, those are things that are not allowed in their project’ (SGA-9), with concerns as to how funding is frequently tied to narrowly defined outputs, disease-specific targets, or time-limited deliverables, with little room for contextual adaptation or responsiveness—‘They will say, ‘we [donors] give the money to you, but we also decide how you're going to spend the money, and we'll audit how you use the money’…but maybe we should be telling them what we need…’ (GA-1). This illustrates the imbalance of influence inherent in financing when the power to define priorities is concentrated in the hands of external actors, sometimes leading to the creation of infrastructure or expansion of services that cannot be sustained once project funding ends—‘…caused the fragmentation in our system…TB had their own supply chain, had its own healthcare workers. And it's actually causing competition at the local level, as opposed to bringing them together to have like a full operational primary health care service’ (SGA-5).

As donor-driven imperatives override locally preferred longer-term system-strengthening approaches, health service providers at the national and sub-national respond to donor-driven reporting requirements and align their activities with narrowly defined programmatic goals; doing so from a place of **self-preservation,** which in turn reinforces health security norms and practices -‘They expect a lot of documentation… it can’t be a budget for infectious disease, it has to be a budget only for TB… It’s not a very sustainable or even realistic type of output they request from the system’ (SGA-5). These administrative demands, combined with the incentive to prioritize donor-funded areas, pull attention and resources away from other pressing local needs: ‘You [health service provider/the government] tend to prioritize donor projects because there's funding… but then we deprioritize other diseases for example’ (SGA-7). At the same time, there were hints that donor funding has positive effects, such as enabling specific programs to reach their targets or providing resources that might not otherwise be available or sustainable, especially in relation to access to services.

During the COVID-19 pandemic, over 350 diagnostic facilities were established, expanding the country’s capacity for in-country testing. Genomic sequencing infrastructure and disease surveillance systems were also developed, largely with donor support. However, these initiatives were described as being rigid, narrowly targeted COVID-19-specific capabilities which lacked the flexibility to be integrated into the already existing system and was unable to pivot or repurposed when needed: ‘So I think we still have a hangover with things like biosurveillance, like genomic surveillance, where can we apply this capability next?’ (GA-9). This places health service providers in a donor-driven cycle of work in which, for example, they became the subject of post-pandemic donor funding as a ‘mop-up’ exercise to repurpose such capacities and capabilities post COVID-19. Even when technical support is provided to enhance existing areas of capability, there is a perception that the support often reinforces verticality, with funding also being directed at programs that are already showing success or offering potential for donor attribution. That is, donors can be seen as ‘buying into’ successful initiatives to reap reputational gains, a consequence of donor efforts at self-preservation, in a cycle in which funding is aimed at an impression of impact—‘So these have been our issues with like a programmatic versus a health system perspective, like maybe like giving TB specific services and X ray vouchers because they can say that there is x 1000 or million Filipinos who benefited as opposed to like a more health system strengthening activities’ (SGA-5).

### Institutionalizing security norms

One pathway through which security language and framing get embedded into routine governance was the whole-of-government approach to pandemic preparedness and response, which participants linked explicitly to the entry of the military into public health domains. While often framed as an efficient and coordinated response mechanism, this approach also reflects deeper ideological tendencies to conflate responses to health emergencies with security and militarized governance: ‘The previous president placed the military above all else, so that's how militarized he was… He prioritized military hospitals for improvement… before him, we didn’t have an MRI machine’ (GA-11). This example shows how security framings legitimized and normalized the reallocation of health resources towards military institutions, which privileges military infrastructure even in civilian health settings. There are also efforts by the DOH to position itself to normalize such efforts. In one example, the DOH was described as leading health security training for the National Defence College, including proposals to embed health security modules in the curriculums as one participant explained, ‘DOH personnel… have been providing lectures… and there have been talks to change the curriculum… to include a module on health security’ (GA-11).

In its institutionalization, the term ‘security’ was described as being interpreted through a narrow, militarized lens, equating health security with armed personnel or law enforcement mandates: ‘Word for word, like security means military personnel, or security means that the military sector or the law enforcement sector….if you're a carpenter, or if you have a hammer…you use a hammer for all of your products’ (GA-1). This conflation of health with security was described as affecting implementation: once health is framed in securitized terms, military actors were seen as logical and necessary implementers. Crucially, the institutionalization of security norms did not occur in isolation from broader questions of equity and marginalization. Several interviewees drew attention to how elite groups benefited from securitized health interventions, while marginalized populations, particularly those living in poverty, were subjected to punitive or exclusionary practices: ‘I think it was very excessive to the plight of our poor countrymen because, for example, the directive to stay home and stay inside. That's not a very easy thing to do for families who are staying in a shoebox’ (GA-3) This was particularly evident in the ways that pandemic lockdowns were enforced in poor communities, often described as an extension of the war on drugs, which had already criminalized poverty in the eyes of the state: ‘I think that's the timing that when COVID hit, the war on drugs was viewed… the lockdowns were viewed as an extension of the drug war’ (GA-11).

The U.S. military system appears to be a key reference point for how the Philippines structures its own defense and security-related health initiatives. Shaped by colonial legacies, this influence was seen as extending beyond ideology into institutional practice: ‘Most of our doctrine is based on the American system. I hate to say it, but we literally copy-pasted everything from them and adapted it for local implementation’ (GA-11). This relationship was also described as shaping decisions around infrastructure and service delivery, particularly where U.S. military presence overlaps with development assistance. For example, one official described how the selection of a site for a new laboratory appeared to be based not on health needs but also proximity to a U.S. military base: ‘I don't know why Region 10 was prioritized, except maybe because the presence of the United States military in Cagayan de Oro… for the ‘one health laboratory’, which is a US grant, they wanted to have a new building for this’ (SGA-4).

## DISCUSSION

This study offers insights into how, why, and for whom health security framing functions across different governance levels and institutional contexts within the Philippines (Pawson and Tilley 1997). It situates the Philippine experience within wider global health governance structures, illustrating how health security, as both a frame and a set of practices, reinforces existing power asymmetries in global health while introducing new configurations of power, responsibility, and influence. By emphasizing the position and the role of the actors—international agencies, national governments, sub-national authorities, and health service providers—not just as implementers of policy, but as active agents who interpret, negotiate, and sometimes resist the framing and practices of health security, we highlight that outcomes are not solely determined by mechanisms or how they manifest as policy, but also by who is enacting them, for what reason, and from which position. These actor roles are dynamic and, in some cases, interchangeable. For example, a national government may adopt donor (Actor 1) framings of health security (as Actor 2) but also reinterpret, enact, or even enforce these priorities within its borders (as Actor 1), thereby shaping how sub-national actors respond (Actor 2). Health service providers at the frontline of policy and program implementation, service users, and the wider community (Actor 3) bear the consequences of decisions made far upstream.

Our findings build on other studies revealing that centralization of governance, at the international level, enabled by technical and financial assistance, (re)-surfaces power asymmetries, as countries with geopolitical clout (e.g. the United States) dictate what is funded and prioritized under the guise of development or partnership, in turn setting norms. For example, Kentikelenis et al. (2023) provide a theoretical nuance reflected in our findings, ‘hegemonic powers’ such as the United States through the WHO shape which frameworks become dominant, and highlight how epistemic authority, for example, when reinforcing agendas aligned with security interests, is exercised by powerful states and institutional actors that steer global norms (Kentikelenis et al. 2023). This dynamic is reflected in our findings, which show that framing health in terms of security affects how priorities are set and resources allocated, regardless of whether national counterparts are fully aware of the underlying agendas set by external entities. The priorities, assumptions, and funding conditions established by powerful states and institutions shape what is deemed fundable or urgent. The agreement with external agendas is not necessarily a passive alignment, and that it can be strategic from the side of the national and sub-national actors; for example, national actors might align for international credibility (self-preservation and self-reliance), and sub-national actors may be reinforcing such dynamics through self-preservation merely to safeguard their own professional positions (Cerny 2010, Baru and Mohan 2018, Holst 2020, Blunt 2022, Sparke and Williams 2022).

At the same time, other geopolitical actors are expanding their influence. While much of the literature has documented the role of the United States in defining such norms and standards (Chorev 2013), other studies, including Benabdallah (2020), explore the rise of China in the foreign policy and global health arena, with a focus on African nations. Through investments in health infrastructure, diplomatic and economic goals are broadened, and we see parallels in the Philippines, where China is seen to deploy health-related resources as instruments of soft power, embedding itself in health governance structures to advance its national interests (Benabdallah 2020).

Our findings show how data plays a key role in legitimizing externally driven priorities—‘external’ not only in being a ‘foreign’, international entity, but also in being a ‘central’, powerful entity that may be domestic but removed from actor 3s’ realities. For example, where the Philippines is required to report on core capacities and indicators, the DOH may feel compelled to ‘perform’ according to these benchmarks, using data to demonstrate competence and alignment, which could be counterproductive to the aim of ensuring certain health capacities. This finding reflects that the dominance of metrics and indicators in global health turns data into a tool of governance, encouraging the need to align with central expectations, which may sideline priorities that do not easily fit into measurable frameworks (Merry 2011, Adams 2016). Secondly, as other studies have shown, the tendency to focus on collecting and reporting surveillance data for specific diseases diverts resources and attention from the social and structural determinants of health, and over-focuses responses towards biomedical solutions such as vaccines and diagnostics (Taylor 2016, Honigsbaum 2017, Holst 2020, Hapal 2021, Holst and van de Pas 2023, Silva and Smith 2023, Russell et al. 2024). While these tools are essential, they often come at the expense of addressing the structural and social conditions that produce vulnerability in the first place (Quintos 2020, Hapal 2021, Holst and Razum 2022, Hecita et al. 2023). While our study explored such findings in relation to health security, similar dynamics may also be examined beyond health security or acute crises, such as pandemics. The processes may reflect broader patterns in global health. In particular, donor-driven frameworks and technical assistance mechanisms reproduce long-standing hierarchies of influence. These dynamics suggest that what appeared as health security governance in the context of this study is also emblematic of the broader political economy of global health.

Our findings also hint at a degree of strategic awareness by the Philippine government of the ‘risks’ of sharing data internationally—even though health security promises to deliver on international cooperation and coordination, this is not without its tensions (McInnes and Lee 2006, Davies 2017, Gerami et al. 2021). This is reflected in South Africa’s experience of travel bans after reporting a new COVID-19 variant, (Jackson et al. 2022) or Indonesia’s assertion of ‘viral sovereignty’ in withholding influenza samples, the Philippines too has demonstrated a level of caution in navigating the politics of data sharing by seeking to exercise agency, protect national interests and resist potential unequal and inequitable consequences of transparency (Elbe and Voelkner 2014, Halabi and Katz 2020, Elbe 2022).

At the country level, national governments also appear to reassert control over health decision-making, which overrides devolved autonomous structures. While other studies indicate that national governments often provide support to fill gaps that are not otherwise filled sub-nationally or locally (Liwanag and Wyss 2019), our findings suggest that in the context of ‘health security’ implementation, local governments are either directed to follow national technical guidance or instructed on how to work with international partners. We can, in turn, infer that the sub-national governments themselves may reinforce centralized practices, particularly when operating from a position of self-preservation or a desire to appear self-reliant, often to the detriment of more grounded, context-specific knowledge held by the local government units (Abimbola et al. 2019).

In the Philippines, the downstream effects of securitized and donor-driven programming were particularly visible at the implementation level. Health service providers, often the bridge between policy and practice, face multiple burdens—navigating donor reporting requirements, implementing vertical programs, and working within systems that lack flexibility and agility to respond to their context-specific needs, as they are often configured to react to external metrics (Kruger 2021). Other studies have similarly highlighted that donor funding imposes conditionalities that distort local priorities and increase bureaucratic burdens (Lakoff 2010, Mwisongo and Nabyonga-Orem 2016). This echoes broader critiques in the literature that link health security agendas to the proliferation of vertical or short-term programs at the expense of system-wide strengthening (Rushton 2011, Wenham 2019). This pattern is particularly visible in the context of HIV/AIDS, which has long been the subject of securitization discussions that have, over time, reinforced donor-dependency and the inability to integrate into national and sub-national health systems, as they continue to remain ‘programs’ (Elbe 2006, Kapilashrami and McPake 2013).

The institutionalization of security norms in public health governance—especially during emergencies—in the Philippines occurs through a mix of political ideology, international influence, and discursive reframing (Capuno and Panganiban 2012, Reid 2016, Hapal 2021, Candelaria and Talamayan 2023). Therefore, ‘security’ is not just about terminology, but about how ideas become embedded in structures and roles. As Hapal (2021) has demonstrated, the security sector in the form of the police and military has become more normalized, not necessarily because it is effective in public health terms, but because it aligns with broader logics of control and order (Hapal 2021, Real 2023, Holst and Razum 2022). While militarization and securitization are not synonymous, militarization can be one of the most visible manifestations of a securitized approach to health, as reflected in the Philippines. Our findings suggest that as security norms become embedded within institutional frameworks, the focus can shift from a public health response to a security response, and in doing so, these dynamics can reinforce hierarchies between global and local actors, national and subnational authorities, military and civilian institutions, and risk silencing the communities most affected by health emergencies (Candelaria and Talamayan 2023). All of which health security framing perpetuates through a ‘whole-of-government’ approach by ‘promoting security as well-being’. Similar examples of securitization of global health is seen in the work in Uganda during the COVID-19 pandemic, which too, gave rise to authoritarian forms of governance (Parker et al. 2022).

An important limitation of this study is that it did not directly include community members or health service users. As our aim was to understand how health security and securitization affect priorities, we deliberately focused on individuals proximate to decision-making, who could provide insight into how the concept of ‘health security’ shapes priorities, governance and resource allocation, to understand the perspective of those who shape and are directly shaped by these decisions. This choice inevitably limits the scope of the findings to decisions and their implementation. We recommend that future studies focus on exploring the downstream and cumulative effects of such decisions as they are translated into access, trust, and first-hand experience of health service users and community members. We also recommend extending the scope of our study to include whether and how health security policies and practices are becoming embedded in the Philippines’ everyday health governance, thereby adding and refining the outcomes identified and further exploring the role of framing health as security as a tool for agenda setting, as they are tied to the experiences of service users. In addition, future studies may also complement previous scholarship, such as the work of Hapal (2021) and Benton (2017), which primarily focus on the role of the security personnel and military in a post-conflict environment, or in relation to a historically authoritarian government, as seen in the Philippines (Benton 2017, Hapal 2021).

While our study may not capture all potential dimensions of health security’s manifestation in the Philippines, our use of theoretically derived mechanisms means that the study can offer insights, including for other countries, into causal mechanisms not explicitly named by respondents. As the study aims to illustrate the contextual fluidity of the outcomes represented, the mechanism-outcome findings will likely resonate in contexts with similar political and institutional arrangements. The engagement of actors at different levels of governance adds complexity to the discussion of power and agency in health systems under external influence. In addition, the study provides country-level empirical insights that are underrepresented in the health security literature, bringing with it a nuanced understanding of how global frameworks are received and interpreted. The theoretical approach has relevance beyond health security framing, offering a framework for analysing how other globally promoted agendas may be variably interpreted and enacted depending on context, governance structures, and actor relationships.

Based on our findings, we recommend that actors at different levels (actors 2), particularly those involved in disseminating international frameworks and designing guidelines for national and sub-national use, should better understand the roots of health security frameworks and interrogate their motivations. Such actors could ask whose priorities these frameworks serve and whether they align with the diverse needs of the Philippines’ contexts, without being compelled to comply solely for external validation or adopt frameworks wholesale. This is particularly important for actors in international organizations and multilateral agencies who not only design but also disseminate frameworks from their central headquarters to country offices. These actors play an important dual role (moving positions between actor 1 and actor 2)—translating global frameworks into country-level programming while simultaneously representing country realities back to global decision-makers. How they navigate this space can either reinforce existing power asymmetries or help to create openings for more responsive, context-sensitive approaches that question the security motive in global health. Such actors, our findings suggest, should not view health workers and implementers primarily as means for implementing global agendas (e.g. surveillance and reporting), but aim to counter the tendency for centralization by surfacing sub-national and local knowledge and needs in priority setting, to avoid the accumulation of governance at any one level, given especially, that health system governance is inherently and necessarily decentralized (Abimbola et al. 2019, 2024).

## CONCLUSION

In summary, this study highlights that health security is not a neutral framework, but rather actively shapes governance, policy, and practice across different levels of the health system. In the Philippines, health security intersects with historical patterns of international influence and authoritarian governance, which have the potential to result in the centralization of governance authority to higher levels, the shaping of resource allocation and health services in line with externally defined priorities, and the institutionalization of security norms and the involvement of security actors in public health. Such outcomes, in terms of how health security manifests, have multiple and varying effects that are not divorced from economic, social, and political contexts. This means that international organizations, national governments, and sub-national actors who design, disseminate, and implement health security frameworks ought to challenge their automatic dominance and adoption. Recognizing the contexts and the conditions in which priority-setting takes place presents an opportunity for more context-sensitive, equity-oriented public health approaches in global health.

## Supplementary Material

czaf110_Supplementary_Data

## Data Availability

The transcripts of the interviews cannot be made publicly available to maintain confidentiality and to protect the identities of study participants. Excerpts from these transcripts may be shared upon request. All data requests should be communicated to the corresponding author.

## Reflexivity statement

This study was conceived as part of the first author’s PhD research. The research team comprised seven members—three male and four female—of whom four are early- to mid-career researchers. All members originate from the Global South, with three currently based in the Global North, including the first author, who has spent the majority of their time in the Global North and has worked on health programmes where health security frameworks were applied. This work aims to examine how health security framing shapes national and sub-national decision-making, particularly in relation to health service prioritization and programme development. All authors are involved in global health research, policy or implementation in various capacities and have encountered, applied, or observed the use of health security and related frameworks in their work. Their collective experiences—predominantly from the Global South—have influenced how the research was designed, analysed, and interpreted.
