# Correction to: Governing health through security in the Philippines: a realist analysis

**DOI:** 10.1093/heapol/czag023

**Published:** 2026-02-17

This is a correction to: Delaram Akhavein, Lea Elora A Conda, Sary Valenzuela, Percival Ethan Lao, Meru Sheel, Seye Abimbola, Geminn Louis C Apostol, Governing health through security in the Philippines: a realist analysis, *Health Policy and Planning*, 2025; https://doi.org/10.1093/heapol/czaf110

The second bullet point in the ‘Key messages’ section was errored. This has been emended to read: “Framing health as a security issue drives centralization of power both within countries and internationally. Securitized health policies favour surveillance and vertical programs, which struggle to integrate into the health system.” instead of: “Framing health as a security issue drives centralization of power both within countries and internationally. Securitized health policies favour surveillance and vertical programs, which struggle to integrate into the health system and create”.

