# Supplementary material for: Governing health through security in the Philippines: a realist analysis
Source: Health Policy Plan. 2025 Dec 12;41(2):262–74. doi: 10.1093/heapol/czaf110 (PMC12906756; doi:10.1093/heapol/czaf110)
Supplement: czaf110_Supplementary_Data [file czaf110_Supplementary_Data.docx]

| Consolidated criteria for reporting qualitative studies (COREQ): 32-item checklist^1^ | |
| --- | --- |
| Topic | Comments/reported on page number |
| 1. Interviewer/facilitator | 24 interviews were conducted by Delaram Akhavein and 1 interview was conducted by Lea Conda. |
| 1. Credentials | Delaram Akhavein is a PhD researcher. Lea Elora A. Conda and Geminn Louis C. Apostol have MDs and MBAs. Sary Valenzuela and Percival Ethan Lao have MDs. Meru Sheel has a PhD. Seye Abimbola has an MD and a PhD. |
| 1. Occupation | Seye Abimbola and Meru Sheel are both Associate Professors in Sydney School of Public Health, where Delaram Akhavein is a PhD researcher. Geminn Louis C. Apostol, Lea Elora A. Conda, Sary Valenzuela and Percival Ethan Lao are researchers at the Ateneo School of Medicine and Public Health. |
| 1. Gender | Delaram Akhavein and 3 other authors are female, and 3 males |
| 1. Experience and Training | Delaram Akhavein is currently undertaking her PhD where she has undergone training for realist methodology including interviewing, with experience working in global health as a scientist. Lea Elora A. Conda, Sary Valenzuela and Percival Ethan Lao are early-mid career researchers in Public Health. Seye Abimbola, Meru Sheel and Geminn Apostol all have 10+ years of experience in health system research and governance and lead on their own respective research areas, are global public health educators, and serve on various boards and committees in relation to research, education and policies. |
| 1. Relationship established | Before the study commenced, the interviewer had some prior contact with some of the participants through her previous role at an international organisation in the country. Further rapport was established at the start of the interview, when the researcher explained the study, addressed participants’ questions, and obtained informed consent. |
| 1. Participant knowledge of the interviewer | The participants only had knowledge of the interviewer in relation to her previous role as per item 6. When participants were recruited, they were provided with an information leaflet about the study, which also provided some information about the research team. including contact details. Delaram Akhavein and Lea Conda explained their background, and the reason for doing the study prior to the start of the interview. |
| 1. Interviewer characteristics | Please refer to data collection |
| 1. Methodological orientation and Theory | Please refer to methods section + theoretical framing |
| 1. Sample | Participants were identified through the authors’ professional network and participant referrals.  Participants were recruited through purposive and snowball sampling. Please refer to participants and sampling section |
| 1. Method of approach | Phone and mail |
| 1. Sample Size | 25 |
| 1. Non-participation | One participant dropped out of the study on the day of the scheduled interview as they did not feel best placed to speak to the study topic. Additionally, three potential participants did respond to the interview invitation. |
| 1. Setting of Data Collection | Online or at offices of participants upon their request |
| 1. Presence of non-participants | No |
| 1. Description of the sample | Please refer to participants and sampling section, table 2 |
| 1. Interview guide | A semi-structured interview guide was developed to support realist interviews. Rather than a fixed set of questions, the guide was structured around theory-driven prompts to explore contexts, mechanisms, and outcomes, with flexibility to probe further depending on participants’ responses. The guide was piloted with the research team (LC & SV). During data collection, the guide was used iteratively, with emerging themes/theories incorporated to further test and refine the programme theories |
| 1. Repeat interviews | No |
| 1. Audio-/visual recording | We used audio recording to collect the data. |
| 1. Field notes | Field notes were made during the interviews and later added to transcribed files. |
| 1. Duration | The interviews lasted an average of 57 minutes |
| 1. Data Saturation | In line with realist methodology, our aim was not to achieve thematic saturation, but rather to reach sufficient explanatory depth to test, refine, and confirm theories. We conducted 25 interviews across different levels of governance and organisational contexts, and the number was partly determined by practical constraints. However, the research team found that the data had reached theoretical sufficiency in relation to our study aims. |
| 1. Transcripts returned | All participants were given the option to review their transcribed file for comments; however, only one participant opted in for this option.. |
| 1. Number of data coders | 2 |
| 1. Description of the coding tree | Please refer to data analysis and synthesis |
| 1. Derivation of themes | Please refer to page 10 |
| 1. Software | Excel and Word were used to manage the data |
| 1. Participant checking | No |
| 1. Quotations presented | Yes, participant quotations are presented to illustrate findings in the results section. |
| 1. Data and findings consistent | Yes. |
| 1. Clarity of major themes | Yes. These are presented in the 4 major findings section |
| 1. Clarity of minor themes | Yes. Minor themes were presented alongside major themes when the overarching findings were related. |

1. Tong A, Sainsbury P, Craig J. Consolidated criteria for reporting qualitative research (COREQ): a 32-item checklist for interviews and focus groups. *International journal for quality in health care : journal of the International Society for Quality in Health Care / ISQua* 2007;19(6):349-57. doi: 10.1093/intqhc/mzm042
